# Heterologous rhamnolipid biosynthesis by *P. putida* KT2440 on bio-oil derived small organic acids and fractions

**DOI:** 10.1186/s13568-019-0804-7

**Published:** 2019-05-31

**Authors:** Stefanie Arnold, Marius Henkel, Janina Wanger, Andreas Wittgens, Frank Rosenau, Rudolf Hausmann

**Affiliations:** 1 Department of Bioprocess Engineering (150k), Institute of Food Science and Biotechnology, University of Hohenheim, Fruwirthstr. 12, 70599 Stuttgart, Germany; 2 Institute for Pharmaceutical Biotechnology, Ulm University, Albert-Einstein-Allee 11, 89081 Ulm, Germany; 3 Ulm Center for Peptide Pharmaceuticals (U-PEP), Ulm University, Albert-Einstein-Allee 11, 89081 Ulm, Germany; 4 Department Synthesis of Macromolecules, Max-Planck-Institute for Polymer Research Mainz, Ackermannweg 10, 55128 Mainz, Germany

**Keywords:** Rhamnolipid, Bio-oil, Pyrolysis, Lignocellulosic biomass, Bioeconomy, Biosurfactant

## Abstract

In many cases in industrial biotechnology, substrate costs make up a major part of the overall production costs. One strategy to achieve more cost-efficient processes in general is to exploit cheaper sources of substrate. Small organic acids derived from fast pyrolysis of lignocellulosic biomass represent a significant proportion of microbially accessible carbon in bio-oil. However, using bio-oil for microbial cultivation is a highly challenging task due to its strong adverse effects on microbial growth as well as its complex composition. In this study, the suitability of bio-oil as a substrate for industrial biotechnology was investigated with special focus on organic acids. For this purpose, using the example of the genetically engineered, non-pathogenic bacterium *Pseudomonas putida* KT2440 producing mono-rhamnolipids, cultivation on small organic acids derived from fast pyrolysis of lignocellulosic biomass, as well as on bio-oil fractions, was investigated and evaluated. As biosurfactants, rhamnolipids represent a potential bulk product of industrial biotechnology where substitution of traditional carbon sources is of conceivable interest. Results suggest that maximum achievable productivities as well as substrate-to-biomass yields are in a comparable range for glucose, acetate, as well as the mixture of acetate, formate and propionate. Similar yields were obtained for a pretreated bio-oil fraction, which was used as reference real raw material, although with significantly lower titers. As such, the reported process constitutes a proof-of-principle for using bio-oil as a potential cost-effective alternative carbon source in a future bio-based economy.

## Introduction

**Fig. 1:**
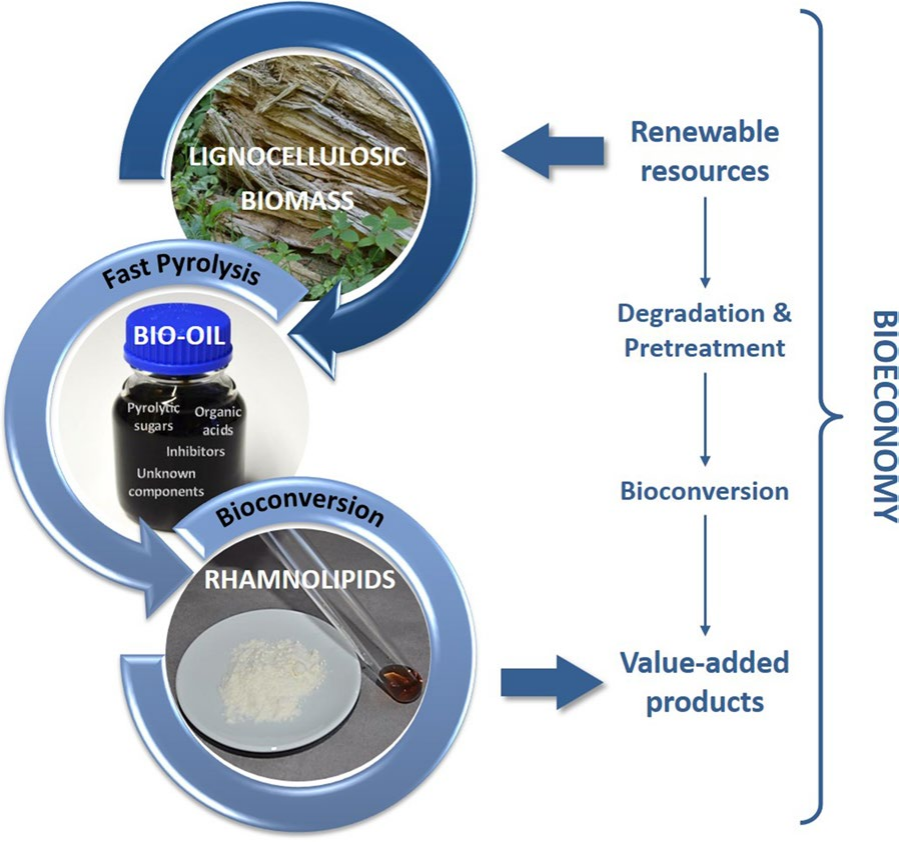
Bio-oil in the value chain of bioeconomy from renewable resources towards value-added products. Envisioned path of conversion from lignocellulosic biomass to rhamnolipid biosurfactants by pyrolysis intermediate steps for decomposition

The establishment of alternative feedstocks as sources of carbon for industrial biotechnology is a key goal to achieve cost-efficient and economical bio-processes. A general competition between food and biotechnology has placed lignocellulosic biomass and other related carbon sources into the focus of attention as renewable and sustainable raw materials. As such, these substrates hold a significant economic and ecologic potential for industrial biotechnology. Lignocellulosic biomass mainly consists of cellulose, hemicellulose, and lignin. For its utilization as carbon sources in biotechnological processes a prior degradation step is necessary. A promising degradation method is fast pyrolysis, which converts lignocellulosic biomass in the absence of oxygen into bio-oil (Fig. 1). Bio-oil mainly consists of water, pyrolytic sugars, small organic acids, phenolic compounds, alcohols, furans, aldehydes and ketones (Piskorz et al. 1988; Mohan et al. 2006; Arnold et al. 2017). Beside pyrolytic sugars, small organic acids such as acetic acid, formic acid and propionic acid are of special interest for biotechnological processes (Prosen et al. 1993; Lian et al. 2010, 2012, 2013; Layton et al. 2011; Linger et al. 2016).

Due to the fact that bio-oil is a complex mixture composed of hundreds of compounds, microorganisms have to be found which are able to metabolize pyrolytic carbon sources such as pyrolytic sugars and small organic acids, as well as tolerate inhibitory compounds present in bio-oil. As previous studies emphasized, the Gram-negative, non-pathogenic and organic solvent tolerant soil bacterium *Pseudomonas putida* KT2440 is a promising candidate for bioconversion of bio-oil (Khiyami et al. 2005; Linger et al. 2016; Arnold et al. 2018). Furthermore, this strain is used as a host for heterologous gene expression for different biotechnological purposes (Dammeyer et al. 2011; Wittgens et al. 2011; Nikel and de Lorenzo 2014; Beuker et al. 2016a).

One interesting example of a potential bulk products of white biotechnology which can be produced on lignocellulose are rhamnolipid biosurfactants. Rhamnolipids are surface-active glycolipids which consist of one or two l-rhamnose units which are linked to one or two hydroxy fatty acids. Rhamnolipids are mainly known to be produced by the opportunistic pathogen *Pseudomonas aeruginosa*. Therefore, research in the last decade has addressed rhamnolipid production in nonpathogenic heterologous production hosts (Ochsner et al. 1995; Cha et al. 2008; Wittgens et al. 2011, 2017, 2018; Tiso et al. 2016).

This study describes the heterologous production of mono-rhamnolipids on small organic acids derived from fast pyrolysis of lignocellulosic biomass, as well as on bio-oil fractions by using a genetically engineered *P. putida* KT2440 strain. Both, growth behavior of the engineered *P. putid*a KT 2440 strain and its simultaneous production of rhamnolipids were investigated during cultivation experiments on acetate, formate and propionate as sole carbon sources, on mixtures thereof, as well as on two different bio-oil fractions, to evaluate the potential of bio-oil as an alternative carbon source for heterologous production of rhamnolipid biosurfactants. As such, the investigated system provides a proof-of-principle for using bio-oil as a potential cost-effective alternative carbon source using rhamnolipid biosurfactants as an example for a value-added product (Fig. 1).

## Materials and methods

### Chemicals and standards

All chemicals used in the current study were either acquired from Carl Roth GmbH (Karlsruhe, Germany) or Sigma-Aldrich (Munich, Germany) if not mentioned otherwise. Mono-rhamnolipid (Rha-C_10_-C_10_) standard was obtained from Sigma-Aldrich Chemie GmbH (Munich, Germany) and rhamnolipid standard as mixture of mono- and di-rhamnolipid from Jeneil Biotech Inc. (Saukville, WI, USA).

### Strain and plasmid

A genetically engineered *P.* *putida* KT2440 strain carrying plasmid pSynPro8oT producing mono-rhamnolipids was used for all cultivation experiments. The plasmid harbors genes *rhlAB* required for rhamnolipid biosynthesis as well as a tetracycline selection marker (Beuker et al. 2016a).

### Media and cultivation conditions

Conditions of fast-pyrolysis, source and applied setup as well as obtained side-streams are described in Arnold et al. (2018). Cultivations for rhamnolipid production were performed as described by Beuker et al. (2016b).

*Pseudomonas putida* KT2440 pSynPro8oT_rhlAB was first incubated in 25 mL LB medium (5 g/L yeast extract, 10 g/L tryptone, 10 g/L NaCl; pH 7.0) containing tetracycline (end concentration 20 mg/L) at 30 °C and 120 rpm.

Growth experiments were carried out in 500 mL baffled shake flasks filled 50 mL of adapted Wilm’s KP_i_ medium (Wilms et al. 2001) (6.58 g/L K_2_HPO_4_, 1.64 g/L KH_2_PO_4_, 5 g/L (NH_4_)_2_SO_4_, 0.5 g/L NH_4_Cl, 2 g/L Na_2_SO_4_, 0.5 g/L MgSO_4_·7H_2_O, 0.05 g/L Thiamin HCl, 3 mL/L trace element solution (trace element solution: 0.18 g/L ZnSO_4_·7H_2_O, 0.16 g/L CuSO_4_·5H_2_O, 0.1 g/L MnSO_4_·H_2_O, 13.9 g/L FeCl_3_·6H_2_O, 10.05 g/L EDTA Titriplex III, 0.18 g/L CoCl_2_·6H_2_O, 0.662 g/L CaCl_2_·2H_2_O). Tetracycline was added to the media to an end concentration of 20 mg/L. Different concentrations of glucose, acetate, formate, propionate, mixtures of small organic acids, or pretreated bio-oil fractions [organic condensate after solid phase extraction (OC_SPE_) and aqueous condensate after solid phase extraction (AC_SPE_)] were added to the medium as carbon source as described previously (Arnold et al. 2018). The main culture medium was inoculated with a starting optical density at 600 nm (OD_600_) of 0.1 using cells washed in 0.9% NaCl solution.

### Analytical methods

Cell growth was monitored by measuring the optical density at λ = 600 nm (OD_600_) using a spectrophotometer (UV-3100 PC, VWR GmbH, Darmstadt, Germany). Consumption of glucose and acetate was determined from the supernatant samples using d-glucose, respectively acetate assay kits (Enztech yellow line, R-Biopharm AG, Darmstadt, Germany) following the manufacturers’ instructions. Rhamnolipid determination was performed as described previously (Horlamus et al. 2019). All graphical and regression analysis for production rates and yield coefficients was performed using scientific graphing and data analysis software (SigmaPlot 13.0, Systat Software Inc., San Jose, CA). If applicable, four parameter logistic fits for biomass and rhamnolipid concentration were used for calculation (Henkel et al. 2014).

## Results

**Fig. 2:**
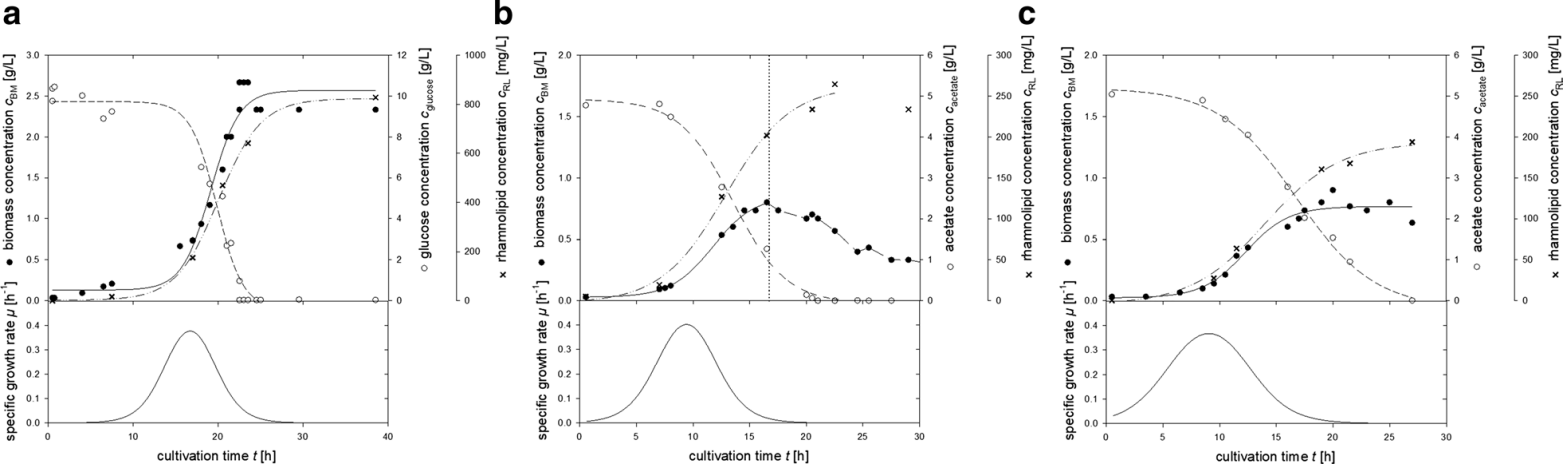
Time-course of biomass, substrate (glucose or acetate) and mono-rhamnolipid concentration during cultivation of *P. putida* KT2440 carrying plasmid pSynPro8oT_rhlAB on **a** 10 g/L glucose, **b** 5 g/L acetate and on **c** a mixture of small organic acids [5 g/L acetate + 1 g/L formate + 1 g/L propionate (= AFP)] as sole carbon source. Data is presented along with respective specific growth rates

Cultivations of the recombinant strain *P. putida* KT2440 pSynPro8oT_rhlAB were performed on glucose, acetate, and mixtures of small organic acids mainly present in bio-oil (Fig. 2). During the time course of cultivation biomass formation, substrate consumption, rhamnolipid production, as well as specific growth rate (Fig. 2—bottom graph) was investigated for (a) 10 g/L glucose, (b) 5 g/L acetate, and (c) 5 g/L acetate + 1 g/L formate + 1 g/L propionate (AFP). Similar growth rates of approximately 0.4 h^−1^ were achieved and rhamnolipid production was detected in all three cultivation experiments, although with different titers. For further insight into production capacity, yields and potential inhibitory effects additional cultivations were performed at different concentrations of organic acids and mixtures thereof (Table 1).

**Table 1:** Summary of cultivation parameters

Carbon source (CS)	*c*_CS_ (g/L)	Growth	*c*_BM_^max^ (g/L)	*µ*^max^ (h^−1^)	*Y*_X|S_^max^ (g/g)	Y_P|X_ (g/g)	*c*_RL_^max^ (mg/L)	*q*_RL_^max^ (mg/g/h)	*q*_RL_^avg^ (mg/g/h)	References
Sunflower oil										
* P. aeruginosa* wt, different strains	Excess	✔	9.0–19.0	0.09–0.18	n.d.	0.9–3.1	8.0–38.0 × 10^3^	60–190	10–40	Müller et al. (2011)
Glucose										
*P. aeruginosa* wt, different strains	20–40	✔	2.4–7.7	n.d.	n.d.	0.3–2.3	0.2–6.1 × 10^3^	n.d.	12–54	Henkel et al. (2016)
*P. putida* KT2440 pSynpro8oT_rhlAB	Excess	✔	23.6	0.44	0.24–0.33	n.d.	14.9 × 10^3^	n.d.	18–24	Beuker et al. (2016a, b)
*P. putida* KT2440 pSynpro8oT_rhlAB	10	✔	2.67	0.38	0.26	0.61	827.7	71.9	42.4	This study
Small organic acids										
Acetate (A)	1	✔	0.20	0.38	0.24	0.76	88.5	74.8	52.7	This study
	3	✔	0.63	0.52	0.23	0.57	191.2	66.7	58.6	
	5	✔	0.80	0.40	0.21	0.62	264.5	62.4	39.2	
	10	✔	0.70	0.15	0.12	0.72	264.2	43.7	23.5	
Formate (F)	1	✖	0	0	n.d.	0	0	0	0	
	3	✖	0	0	n.d.	0	0	0	0	
	5	✖	0	0	n.d.	0	0	0	0	
Propionate (P)	1	✔	0.23	0.25	n.d.	0.90	119.4	74.7	62.4	
	3	✔	0.73	0.24	n.d.	0.47	184.0	49.8	53.9	
	5	✔	0.67	0.16	n.d.	0.61	212.3	19.9	24.7	
	10	✔	2.57	0.21	n.d.	0.07	93.7	n.a.	n.a.	
Mixtures of small organic acids										
AF	5 + 1	✔	0.63	0.49	0.18	0.56	185.9	56.9	40.4	
AP	5 + 1	✔	0.87	0.50	0.21	0.53	239.7	80.6	40.8	
AFP	5 + 1 + 1	✔	0.90	0.37	0.25	0.41	193.9	72.1	26.6	
Bio-oil fractions										
OC_SPE_	1^a^	✔	0.27	0.39	0.24	0.48	72.5	28.4	n.a.	
	3^a^	✔	0.37	0.20	0.10	0.43	89.4	2.8	1.8	
	5^a^	✖	0	0	n.a.	0	0	0	0	
AC_SPE_	1^a^	✖	0	0	n.a.	0	0	0	0	
	3^a^	✖	0	0	n.a.	0	0	0	0	
	5^a^	✖	0	0	n.a.	0	0	0	0	

^a^The bio-oil fractions were added in three different concentrations adjusted according to acetate concentration

✔ growth, ✖ no growth observed, *n.a.* not applicable, *n.d.* not determined

To compare acetate with glucose different acetate concentrations from 1 to 10 g/L were applied. The maximum concentration range was chosen depending on typical concentrations in bio-oil resulting from different applied raw material and pyrolysis process conditions. An acetate concentration of 10 g/L approximately corresponds to a 1:10 dilution of average bio-oil composition, which, due to its very high viscosity and inhibitory components, is a reasonable working dilution. Results from cultivations up to 5 g/L acetate revealed that similar growth rates (µ = 0.38–0.52 h^−1^) and yields (Y_X|S_ = 0.21–0.24 and Y_P|X_ = 0.57–0.76) were reached when compared to cultivations on glucose from this study (µ = 0.38, Y_X|S_ = 0.26, Y_P|X_ = 0.61) as well as data from literature (Table 1). While growth rate is significantly reduced to 0.15 h^−1^ at acetate concentrations of 10 g/L, results suggest that an inhibitory effect on growth might not necessarily correlate with an inhibition of rhamnolipid biosynthesis which, at a similar yield coefficient Y_P|X_ of 0.72 compared to 0.62, reaches a similar titer above 250 mg/L, both at 5 g/L and 10 g/L acetate. When using acetate as a sole source of carbon, a decrease in biomass can be observed at a certain biomass concentration reducing biomass from 0.8 g/L below 0.5 g/L (Fig. 2b).

Cultivations with formate as sole source of carbon revealed that with the applied system no metabolization of formate was observed. To further assess its inhibitory effect, formate was further used simultaneously with acetate and acetate plus propionate (AP), respectively. A concentration of 1 g/L formate was chosen, which corresponds to a 1:10 dilution of average bio-oil composition. At the applied concentration, formate does not seem to have a negative effect on either growth or product formation (Table 1).

Growth rates were reduced to approximately 50% (µ = 0.16–0.25 h^−1^) compared to acetate when using propionate in different concentrations as sole carbon source. An adverse effect on rhamnolipid biosynthesis was observed at propionate concentration between 3 and 5 g/L (Table 1). Despite comparably high biomass production on 10 g/L propionate with a maximal biomass of 2.57 g/L, the maximal rhamnolipid concentration was reduced from 212.3 mg/L for 5 g/L propionate to 93.7 mg/L for 10 g/L propionate. Propionate was further studied as an additional carbon source with acetate and acetate plus formate (AF), respectively. The concentration of 1 g/L propionate was applied, which also corresponds approximately the concentration of a 1:10 diluted average bio-oil composition. Results suggest that, in the applied concentrations, propionate, comparable to formate, has no negative effect on growth and product formation (Table 1).

Growth and rhamnolipid production was also possible on mixtures of small organic acids with a corresponding concentration of a 1:10 diluted average bio-oil composition. Formate and propionate had no observable negative effect on either growth or rhamnolipid formation.

**Fig. 3:**
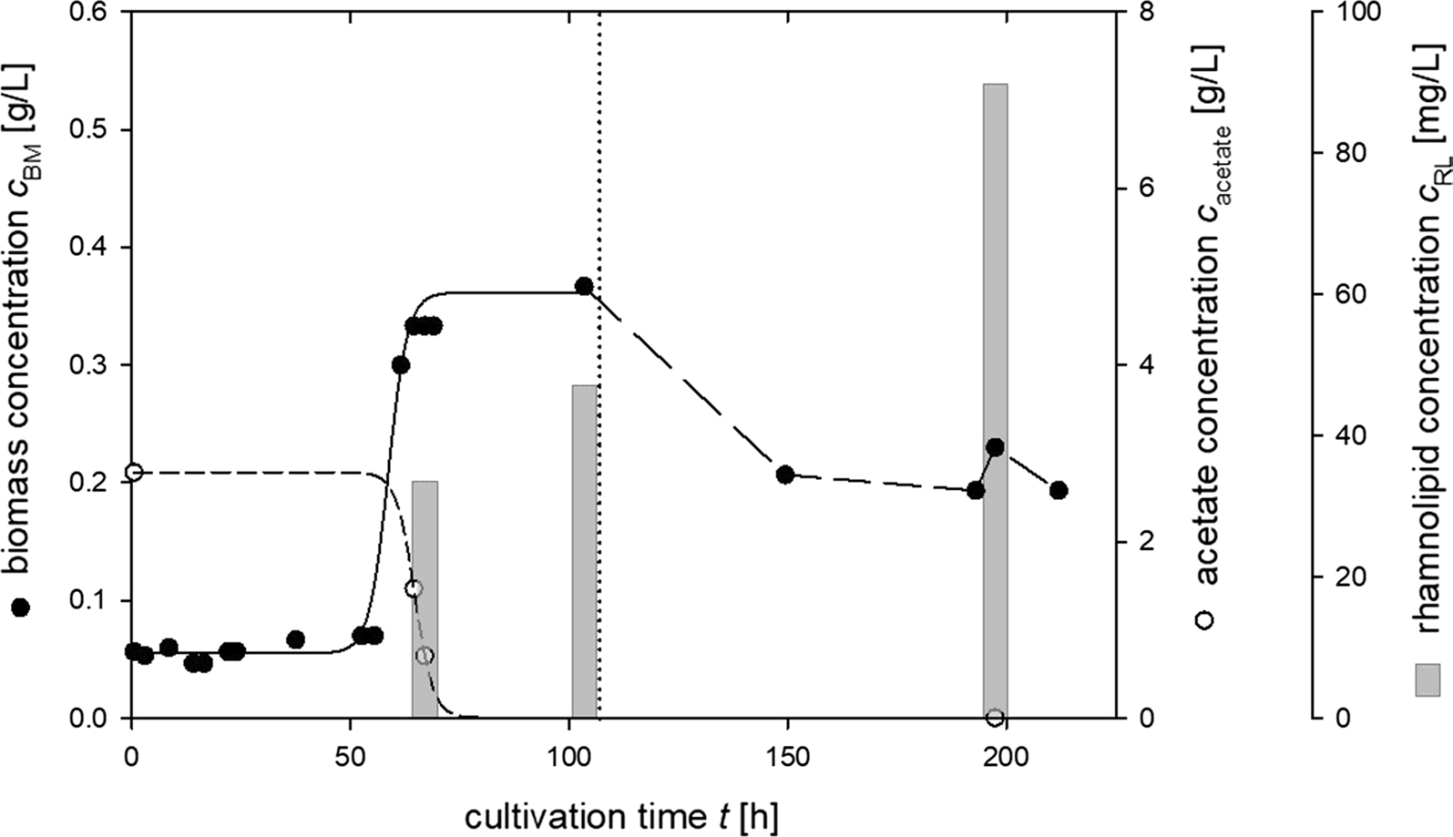
Time-course of biomass (filled circles), acetate (empty circles) and mono-rhamnolipid concentration (bars) during cultivation of *P. putida* KT2440 carrying plasmid pSynPro8oT_rhlAB on organic condensate of bio-oil pyrolysis process after solid phase extraction (OC_SPE_) containing 3 g/L acetate

Furthermore, growth and rhamnolipid production was investigated using pretreated bio-oil fractions as sole carbon sources. The bio-oil fractions OC_SPE_ and AC_SPE_ were added in three different concentrations of acetate up to 5 g/L adjusted as described by Arnold et al. (2018). While growth was detectable on OC_SPE_ with concentrations of up to 3 g/L (Fig. 3), a significant inhibitory effect on growth is already observed at this concentration, resulting in a reduction of specific growth rate from 0.39 h^−1^ to almost half the value of 0.20 h^−1^. In contrast, no biomass formation was observed when AC_SPE_ was used as sole carbon source. To reach a final concentration of acetate of 3 g/L in OC_SPE_, an approximate 1:35 dilution of average bio-oil would be required. This is a significantly higher dilution compared to application of artificial substrate mixtures, however, is explained by a high number and amount of inhibitory and unknown components in bio-oil. Compared to cultivations on acetate this further shows an inhibitory effect on rhamnolipid biosynthesis, where addition of 3 g/L acetate in OC_SPE_ does not significantly increase rhamnolipid titers of 72.5 mg/L observed at 1 g/L acetate in OC_SPE_.

## Discussion

In summary, the results suggest that acetate represents a potentially suitable carbon source for rhamnolipid production. In direct comparison to rhamnolipid production obtained with the same strain and genetic construct on glucose as a sole source of carbon from this study as well as previous studies (Beuker et al. 2016a, b), both maximum specific rhamnolipid production rate as well as average production rate are generally in a similar range between approx. 45–75 mg/(g h) and 25–45 mg/(g h) (Table 1). It should be noted however that while maximum specific rhamnolipid production rates on glucose and low concentrations of acetate are highly similar [71.9 mg/(g h) on glucose versus 74.8 mg/(g h) on 1 g/L acetate], this is not the case for average production rates, which are approx. half in the study used for comparison with the same biological system (18–24 mg/(g h) versus 42 mg/(g h). This is due to the fact that Beuker et al. (2016a, b) developed a fed-batch process over 3 days which was aimed for high-titer production, and thus not optimized for average or maximum production rates. For reference, high-yield processes with *P. aeruginosa* using plant or vegetable oil are further used for comparison. Obtained titers of up to 38 g/L as well as maximum production rates of up to 190 mg/(g h) are significantly higher than for both glucose as well as organic acids. Average production rates, on the other hand, are on average lower for *P. aeruginosa* using plant or vegetable oil. This is due to the fact that quorum sensing has a major effect on rhamnolipid biosynthesis in wild-type *P. aeruginosa* thus significantly restricting the window of time during cultivation where rhamnolipid production is observed. However, comparing rhamnolipid production of *P. aeruginosa* on glucose, similar ranges are observed for average production rates up to 54 mg/(g h) compared to *P. putida* KT2440.

While the results confirm that rhamnolipid biosynthesis is possible using bio-oil derived carbon sources, for competitiveness on an economic level, several factors would have to be addressed. These factors include modification of the biocatalyst towards higher rhamnolipid productivity, increased metabolic spectrum to metabolize additional components in bio-oil or by engineering for higher tolerance towards inhibitory components. Furthermore, considering the high amount of different substances with potentially inhibitory threshold concentrations, a fed-batch process with continuous addition of substrate may not be feasible due to accumulation of potentially inhibiting or unmetabolized components. Therefore, appropriate techniques for establishing a bioprocess may include investigation of bioreactor systems with biomass retention and flow-through system of medium such as membrane bioreactor constructions, biofilm reactors or perfusion bioreactor systems. In addition, a modification of the applied plasmid-based expression system containing rhamnolipid biosynthesis genes may lead to increased productivity. These modifications may include studies on promoter activity or stable genomic integration. Furthermore, additional renewable sources of acetate or acetate-containing side-streams such as wastewater from cellulose manufacturing (pyroligneous acid) are interesting targets for future investigation.

In this study, it was shown that acetate represents a potentially suitable substrate for biotechnological production of rhamnolipids. Results show that maximum achievable productivities, as well as substrate-to-biomass yields are in a similar range compared to glucose. Furthermore, bio-oil was used in this study as an example for a renewable source of acetate to investigate its application as an alternative substrate for rhamnolipid production. Owing to its very high viscosity as well as complex composition of inhibitory components, bio-oil has to be pretreated and diluted before application for rhamnolipid production. Cultivations with pretreated bio-oil fractions resulted in lower titers with an onset of inhibitory affects at much lower concentrations than for acetate. However, even though bio-oil represents a challenging substrate for rhamnolipid production, it should be noted that maximum production rates of approximately half of different reference processes could be observed. As such, the reported process constitutes a proof-of-principle for using bio-oil as a potential cost-effective alternative carbon source in a future bio-based economy.

## Data Availability

All obtained data have been included into the manuscript. Please turn to the corresponding author for all other requests.

## Abbreviations

OC_SPE_: organic condensate after solid phase extraction
AC_SPE_: aqueous condensate after solid phase extraction
RL: rhamnolipid
AF: acetate + formate
AP: acetate + propionate
AFP: acetate + formate + propionate
